# A Transferable Quantitative Framework for Extracting Engineering-Relevant Descriptors from Biological Protective Surfaces: Intra-Specimen Descriptor Mapping of Five Citrus Peels

**DOI:** 10.3390/biomimetics11070451

**Published:** 2026-06-30

**Authors:** Murat Bengisu, Burcu Akdağ, Fatma Şahmurat, Zehranur Tekin, Kamile Nazan Turhan

**Affiliations:** 1 Department of Industrial Design, Izmir University of Economics, 35330 İzmir, Türkiye; murat.bengisu@ieu.edu.tr; 2 Center for Materials Research, Izmir Institute of Technology, 35430 İzmir, Türkiye; burcuakdag.iue@gmail.com; 3 Design Studies, Izmir University of Economics, 35330 İzmir, Türkiye; 4 Department of Food Engineering, Aksaray University, 68100 Aksaray, Türkiye; fatmasahmurat@aksaray.edu.tr; 5 Department of Food Engineering, Izmir University of Economics, 35330 İzmir, Türkiye; zehranur.gicir@ieu.edu.tr

**Keywords:** biomimetics, bioinspired design, biological protective surfaces, citrus peel, quantitative descriptor framework, SEM image analysis

## Abstract

Citrus peel is examined here as a naturally evolved protective surface, with the goal of developing a transferable quantitative framework for extracting engineering-relevant descriptors from biological protective surfaces and using them as design templates for biomimetic counterparts. A single-specimen-per-species design is adopted to map intra-fruit geometric variation across regions and magnifications; absolute descriptor values are therefore reported as ordinal indicators of inter-species ranking rather than as population means. Five citrus species (lemon, orange, mandarin, grapefruit, and bitter orange) were characterised by mechanical testing (cutting, puncture, and compression; five replicates per fruit), gravimetric peel density and thickness, and scanning electron microscopy (SEM) at 100×–10,000×. The 135-image SEM dataset was processed with an automatic-calibration pipeline performing per-image scale-bar detection, multilevel-Otsu segmentation of albedo air space, cell-bounded surface segment (CBSS) and oil-gland segmentation on flavedo, and grey-level co-occurrence matrix (GLCM) texture analysis with a directional anisotropy index $A_{F}$. Calibration was consistent across all images (FoV × magnification =403,273±410 μm·×, ±0.10%). Principal component analysis separated flavedo and albedo at every magnification (PC1 + PC2 = 84–92%). Within this dataset, grapefruit showed the densest CBSS cover (1072 ${\mathrm{mm}}^{-2}$) together with the highest oil-gland density (2.77 ${\mathrm{mm}}^{-2}$); bitter orange showed the largest CBSS area (23.7 $\mu m^{2}$) and the thickest peel (13.1 mm); mandarin showed the most directionally oriented flavedo film ($A_{F}=0.0885$); and lemon showed the most open albedo ($\varphi_{2D}=36.2\%$). Oil-gland equivalent diameter was essentially invariant (∼45 μm) across the five fruits, while gland density varied 4.4-fold. The structural metrics define a layered descriptor space—a dense isotropic surface relief versus a thick cellular bulk—that supplies two distinct bioinspired-design priors: dense surface films as a structural prior for selective-permeability membranes and layered cellular cores as a prior for impact-absorbing panels. A modified-atmosphere packaging (MAP)-compatible biomimetic film is identified as one downstream design hypothesis requiring direct gas-permeability verification on synthetic membranes.

## 1. Introduction

In morphological terms, citrus species are simple fruits made up of one or several fused carpels; the fruit comprises an outer exocarp (peel), a middle mesocarp and an inner endocarp [1]. The leathery exocarp protects the fruit from impact, ultraviolet radiation, oxidation and dehydration, so that the fruit remains edible by the vector animals that disperse its seeds [2,3]. From an engineering perspective, the exocarp can therefore be regarded as a naturally evolved protective surface that has been refined to balance impact resistance, gas exchange and dehydration control.

The two layers of the peel differ in both composition and structure. The flavedo layer is composed predominantly of cellulose fibres providing strength and damage resistance. Essential oils, paraffin waxes, steroids, triterpenoids, fatty acids, pigments, bitter principles and enzymes fulfil biochemical rather than structural functions [1]. The albedo layer is composed of cellulose fibres, soluble carbohydrates, pectin, protopectin, flavonoids, amino acids and vitamins; pectin contributes to mechanical performance in this layer. The albedo has a markedly lower density than the flavedo because of its spongy nature and the absence of dense oil-gland tissue.

From an engineering perspective, citrus peel can be described as a sandwich structure [3] formed of a thin outer flavedo, a lower-density albedo core and a thin membrane at the transition to the pulp. Both layers are reinforced by vascular bundles, and the whole sandwich behaves as a functionally graded cellular foam. Bührig-Polaczek et al. [4] showed that the foam-like albedo of pomelo has an interconnected pore structure, in contrast to most other plant tissues that are closed-cell or honeycomb-like. Tamer et al. [5] reported ∼50% porosity for fresh orange peel with a bimodal size distribution peaking at 19.8 μm and 7.2 μm. SEM-based reviews have consolidated the use of GLCM texture descriptors and fractal dimension to characterise food surfaces quantitatively [6,7].

This sandwich architecture also defines the mechanical role of the peel. The 2–3 cm thick spongy albedo of pomelo fruits has been shown to dissipate over 90% of the impact energy of fruits falling from up to 15 m [8,9]. Studies of pomelo and other citrus species have served as the basis for biomimetic metal foams and sandwich structures [4]. Once ripe, the fruit depends on an intact peel for microbial protection; any damage exposes the endocarp and triggers decay [8]. In parallel, citrus peels are generated in very large quantities as agro-industrial residues, and their valorisation as biodegradable packaging materials has attracted growing interest [10,11].

These mechanical and structural features have made the peel a recurring reference in biomimetic design. This view of the peel as a naturally engineered protective surface has been formalised in the biomimetics and bioinspired-design literature, with pomelo (*Citrus maxima*) peel acting as a reference system for impact-absorbing foams and sandwich composites [4,8,9]. Comparative quasi-static compression on five citrus species has further described the peel as a functionally graded cellular material with near-zero Poisson ratio, indicating an auxetic-like, energy-dissipating behaviour at the whole-fruit scale [12]. Beyond citrus, comparable hierarchical natural architectures have been examined, including *Ganoderma lucidum* fruiting bodies, which combine a dense outer crust, a porous oriented context and segmented hymenial tubules [13]. Biomimetic packaging films inspired by lotus-leaf surfaces have demonstrated water-contact angles above $150^{\circ }$ and antibacterial efficacy above 95% [14], while postharvest mechanical-handling devices such as soft-shell grippers for cucumbers have been developed to preserve the natural peel structure during transport [15]. Citrus processing residues exceed 50% of the harvested fruit mass and are already used at industrial scale as feedstock for pectin, essential-oil and bioactive recovery [16]. Binder-free thermo-pressed panels of orange peel have been reported with bulk densities around 558 kg/$m^{3}$ and thermal conductivities of 0.065–0.077 W/m·K; this demonstrates the technical feasibility of building macroscopic objects directly from the unmodified peel [17]. Parallel attempts to use mandarin peel powder as filler in biodegradable polymer matrices increase elastic modulus but reduce tensile strength and elongation, with molecular-weight losses attributed to the residual citric acid of the peel [18]; this contrast motivates work on the native peel architecture rather than on ground-peel fillers. Nevertheless, comparative quantitative microstructural data across multiple citrus species, and an explicit translation of these data to a postharvest packaging context, remain limited.

The aim of the present study is to develop a transferable quantitative framework for extracting engineering-relevant descriptors from biological protective surfaces, and to demonstrate its application on the peels of five citrus species. Five citrus species (orange, mandarin, bitter orange, grapefruit, and lemon) are compared in terms of peel mechanical and physical properties. The characterisation is complemented by a quantitative SEM-based microstructural analysis built on per-image calibration, multilevel-Otsu segmentation, GLCM texture descriptors and principal component analysis.

The contribution of the present work to biomimetics is threefold. First, it introduces a reproducible, openly released pipeline that converts SEM micrographs of a biological protective surface into a small set of physically interpretable descriptors (oil-gland density and equivalent diameter, 2D void fraction $\varphi_{2D}$ on the exposed inner-albedo surface, CBSS counts and areas, GLCM texture metrics and a directional anisotropy index $A_{F}$), and that can be applied without modification to other plant or animal protective layers. Second, it reports a comparative descriptor map across five *Citrus* species, identifying which structural axes are conserved across the genus and which vary across species, and so isolating the parameters that a bioinspired counterpart would have to control in order to reproduce a given native function. Third, it groups the species-level descriptors into two distinct bioinspired-design templates derived from the same biological system—a dense, isotropic, surface-film template (grapefruit-type) and a thick, layered, cellular-core template (bitter-orange-type)—providing direct numerical targets for additive-manufactured or polymer-cast biomimetic surrogates. Beyond its mechanical function, early phenomenological studies on intact citrus fruit proposed that the peel may also contribute to creating an internal modified-atmosphere packaging (MAP) like environment that affects postharvest behaviour [19,20]; recent quantitative work has begun to characterise this barrier function in terms of cultivar-dependent peel gas diffusion resistance [21] and peel gas permeance under postharvest treatments [22], within a broader framework of fruit respiration and gas exchange [23]. In parallel, citrus peel constituents and waste streams are being actively explored as feedstock for biodegradable edible films and packaging composites [11,24,25]. Within this context, the present descriptor map is intended to provide quantitative microstructural reference values for biomimetic surface designs; the suggestion that a grapefruit-type surface-film template could be transferred to a synthetic MAP-like coating is treated here as a speculative design hypothesis that would require direct gas-permeability verification on cast or printed surrogates.

## 2. Materials and Methods

### 2.1. Plant Material and Mechanical/Physical Evaluation

Five citrus species (lemon, sweet orange, mandarin, grapefruit, and bitter orange; *C. limon*, *C. sinensis*, *C. reticulata*, *C. paradisi*, and *C. aurantium*) were obtained on the same day from a single local retailer in Izmir, Türkiye. For each species, fruits were drawn from a single retail batch of commercially mature, visually unblemished specimens characteristic of the species in colour, shape and size. For each species, one fruit was used for whole-fruit mechanical testing (five replicate measurements at distinct positions on that fruit), a single additional fruit from the same retail batch was used for SEM imaging, and peel-density values were obtained as the mean over five separate fruits of the same batch. Five mechanical replicates per test were performed at distinct upper, equatorial and lower positions on the single mechanical-test fruit, so the reported force standard deviations quantify within-specimen positional variability. The peel-density standard deviation, computed over five separate fruits of the same batch, quantifies between-fruit variability within that batch; neither standard deviation estimates biological variability across the wider retail population.

Mechanical properties were determined with a Texture Analyser (TA-XTplus, Stable Micro Systems, Godalming, Surrey, UK) equipped with a 50 kg load cell. Compression tests were conducted on whole fruits with a flat 200 mm circular steel plate at 0.02 mm/s to a 16 mm displacement (25 mm for grapefruit). Cutting tests used an extended craft knife affixed to the probe carrier at 1 mm/s. Puncture tests used a 5 mm cylindrical probe at 1 mm/s; the top, middle and bottom of each fruit were tested.

Peel densities were determined gravimetrically on peel samples from five separate fruits per species and reported as the mean. Peel samples with well-defined dimensions were measured with calipers and weighed on a balance with 0.001 g precision; density was calculated as the ratio of mass to volume; albedo densities were measured separately when the albedo could be cleanly detached from the flavedo. In the mandarin specimen, the peel was too thin (∼3.2 mm) to allow clean separation of the two layers without contamination, so the mandarin albedo density was not reported (entered as “–” in Table 1). For specimens where the flavedo and albedo could be cleanly separated, flavedo and albedo thicknesses were measured individually with calipers; the flavedo fraction is reported as $t_{\mathrm{flavedo}}/t_{\mathrm{peel}}$. Scanning electron microscopy used an FEI Quanta-FEG 250 (FEI, Hillsboro, OR, USA) at 100×–10,000×. Peel pieces for SEM were taken from specimens of the same retail batch. Peel pieces were briefly rinsed with water to remove loose surface debris and were then ambient air-dried; no solvent treatment, critical-point drying or freeze-drying step was used. Specimens were mounted on aluminium stubs with double-sided carbon tape and sputter-coated with a gold layer of approximately 5–10 nm before imaging. Per-image acceleration voltage, working distance, detector type and magnification are encoded in the metadata banner of each micrograph and are propagated through the image-analysis pipeline (Section 2.3).

### 2.2. Descriptive Comparison of Mechanical and Physical Properties

Group means and standard deviations are reported as descriptive statistics over the five technical replicates described in Section 2.1. Between-species differences are discussed qualitatively; no between-species inferential test was performed because each species is represented by a single retail batch rather than by an independent biological sample of the wider population. All summaries were computed in Python (v.3.13.9) using scipy.stats.

### 2.3. Quantitative SEM Image Analysis

#### 2.3.1. Image Inventory and Calibration

A total of 135 SEM micrographs were analysed, covering five species (*C. limon*, *C. sinensis*, *C. reticulata*, *C. paradisi*, and *C. aurantium*), two layers (flavedo and albedo) and five magnifications (100×, 250×, 500×, 1000× and 2500×, with selected samples at 5000×–10,000×). Image-level replication ranged from 1 to 4 micrographs per species–layer–magnification cell (mean n=2.3).

Pixel size was calibrated automatically from the embedded scale bar in the bottom metadata banner of each frame. The bar pixel length $L_{i}^{\mathrm{px}}$ was detected as the longest continuous horizontal dark run in the right-most banner cell; the labelled length $L_{i}^{\mu m}$ was encoded in the file name. The per-image pixel size was

(1) $$s_{i}=\frac{L_{i}^{\mu m}}{L_{i}^{\mathrm{px}}}.$$

Detection succeeded on 135/135 images (100%). Internal calibration consistency was verified by computing, for every image, the product of the horizontal field of view and the labelled magnification, which is a microscope constant under correct calibration. The 135 images returned 403,273 ± 410 μm·× (relative spread ±0.10%), confirming consistent calibration across magnifications and species.

#### 2.3.2. Pre-Processing

The bottom 8% of each frame (metadata banner) was cropped; the remainder was converted to 8-bit greyscale and contrast-enhanced by Contrast Limited Adaptive Histogram Equalisation (CLAHE) [26] with clip limit 2.0 and 8×8 tile grid.

#### 2.3.3. Module Assignment by Magnification

Module 2 (flavedo oil-gland and pore segmentation) was applied at 100× and 250×. Module 4 (flavedo cell-bounded surface segmentation, CBSS) was applied at 500×. Module 1 (albedo air-space quantification) was applied at 250× and 500×. Module 3 (GLCM texture and edge density) was applied at every magnification.

#### 2.3.4. Module 1—Albedo 2D Void Fraction from Exposed Inner Surface

Albedo images were partitioned into background, wall and bright-edge classes by multilevel Otsu thresholding [27,28]. The lowest-intensity class was retained as intercellular air space. The areal void fraction is

(2) $$\varphi_{2D}=\frac{\sum_{\left(x,y\right)}⊮\left\{m\left(x,y\right)=1\right\}}{\sum_{\left(x,y\right)}1}\times 100\%,$$

computed on SEM micrographs of the exposed inner albedo surface (i.e., the inner face of the peel after manual separation of the flavedo) and reported as a relative 2D void-area descriptor rather than as true 3D porosity [29]. Because intercellular voids in the spongy albedo intersect the imaged inner surface, $\varphi_{2D}$ acts as a stereological estimator of the underlying 3D void fraction and may differ from volumetric porosity for anisotropic porous media [30,31].

#### 2.3.5. Module 2—Flavedo Oil-Gland and Pore Detection

Candidate dark regions were obtained by adaptive Gaussian thresholding (151 px block, offset 8) applied to a Gaussian-pre-smoothed image (σ=2 px), followed by morphological opening (5 × 5 elliptical kernel) and closing (9 × 9 elliptical kernel) to consolidate the gland regions. For each connected component, area $A_{k}$, perimeter $P_{k}$, equivalent diameter $D_{k}=2\sqrt{A_{k}/\pi }$ and circularity

(3) $$C_{k}=\frac{4\pi A_{k}}{P_{k}^{2}}$$

were measured. A circularity threshold $C_{k}\geq 0.6$ and a minimum area $A_{k}\geq 1000$ $\mu m^{2}$ were applied. The circularity cut-off corresponds to the conventional shape-based criterion for retaining approximately round objects in SEM-based food-microstructure analysis [6,7]: mature citrus oil glands are sub-spherical cavities and project as quasi-circular features on the imaged flavedo surface at the magnifications used here, whereas elongated artefacts (fold lines, scratches, edge fragments) fall below this threshold. The minimum-area cut-off of 1000 $\mu m^{2}$ corresponds to an equivalent circular diameter of approximately 36 μm, which lies below the lower bound of the oil-gland size range reported for citrus pericarp (typically 50–250 μm in diameter [6,7,32]) and therefore retains intact glands while excluding sub-resolution wax pits and small dark spots that are not mature glands. No explicit upper area limit was imposed: the combination of the circularity filter and the border-touching rejection (regions whose bounding box reaches within two pixels of the frame edge) already removes the large irregular artefacts (mounting-tape edges, scratches) that would otherwise dominate the upper tail. Per-frame counts were normalised to ${\mathrm{mm}}^{2}$ of imaged surface (glands · ${\mathrm{mm}}^{-2}$).

#### 2.3.6. Module 3a—GLCM Texture

Intensities were quantised to 32 grey levels. GLCMs P(i,j) were computed at two displacements d∈{1,3} px and four orientations $\theta \in \{0^{\circ },45^{\circ },90^{\circ },135^{\circ }\}$, made symmetric and normalised. Given the per-image pixel sizes derived from the calibration constant (2.68 μm/px at 100×, 1.07 μm/px at 250×, 0.54 μm/px at 500×, 0.27 μm/px at 1000×), d=1 px probes the smallest resolvable structural scale at each magnification and d=3 px probes a 3-fold larger scale, so the two displacements together capture both fine cuticular roughness and coarser CBSS-scale variation without requiring a magnification-specific re-scaling. Haralick contrast, dissimilarity, homogeneity, energy, correlation and entropy [33] were computed and averaged across orientations. The directional anisotropy index for any feature *F* is

(4) $$A_{F}=\frac{\sigma_{\theta }\left(F_{\theta }\right)}{\bar{F}}.$$

GLCM contrast and entropy at 1000× were retained as primary surface-texture descriptors in line with prior food-surface SEM work that has linked GLCM features at this magnification range with texture and mechanical attributes [32,34].

#### 2.3.7. Module 3b—Edge Density

Canny edge detection [35] was applied to the gradient magnitude image with Otsu-derived hysteresis thresholds $T_{\mathrm{low}}=0.5\;T_{\mathrm{Otsu}}$ and $T_{\mathrm{high}}=T_{\mathrm{Otsu}}$. Edge density is the fraction of edge pixels in the cropped frame.

#### 2.3.8. Module 4—Flavedo CBSS Segmentation

At 500×, inverse Otsu thresholding followed by morphological closing was applied; the marker-controlled watershed transform [36] on the Euclidean distance map separated touching segments. Markers were obtained by h-maxima detection (h=2). Segments smaller than 30 px or touching the image border were excluded, and a circularity sanity filter $C_{k}\geq 0.60$ was applied as the default criterion. The $C_{k}\geq 0.60$ threshold rejects the elongated sliver fragments that the watershed step generates along cuticular folds and at touching-segment boundaries: intact cell-scale projections on a smooth flavedo surface are near-isotropic and remain above this cut-off, whereas linear watershed artefacts (single-pixel-wide ridges, partially merged segments) fall below it. The threshold is consistent with shape-based criteria used in SEM-based food-surface segmentation [6,7]. A more permissive threshold ($C_{k}\geq 0.45$) was used for bitter orange and grapefruit, because the broad cuticular folds and locally separated wax sheets of these two species fragment into mildly elongated but still topologically valid segments that the stricter default would discard together with the genuine surface-relief features; the relaxed threshold preserves these features while still excluding single-pixel ridge artefacts. This species-specific adjustment is reported descriptively here and re-examined in the limitations section. The retained segments are referred to as cell-bounded surface segments (CBSS), that is, topographic projections of cell-scale boundaries rather than histologically resolved cells.

#### 2.3.9. Multivariate Analysis

For each magnification, GLCM features, GLCM anisotropy indices and edge density were standardised (z-score) and projected onto two principal components.

#### 2.3.10. Software

The pipeline was implemented in Python (v.3.13.9) using NumPy [37], SciPy (v.1.16.3) [38], scikit-image (v.0.25.2) [39], OpenCV (v.4.13.0.92) [40], scikit-learn (v.1.7.2), matplotlib (v.3.10.6), seaborn (v.0.13.2), and pandas (v.2.3.3). The pipeline was developed and iteratively debugged with the assistance of generative AI coding tools (see Acknowledgments); all algorithmic choices, parameter values and threshold rules were defined, verified and validated by the authors against the raw micrographs, and the full source code is openly released on Zenodo (DOI: 10.5281/zenodo.20350228) so that every step can be independently re-executed.

#### 2.3.11. Statistical Reasoning for SEM Data

The study intentionally prioritises high-resolution intra-specimen structural mapping over population-level biological inference: each species is represented by one fruit imaged densely across four magnifications and three peel regions, rather than by multiple fruits imaged sparsely. This trades species-level statistical power against within-specimen geometric resolution, and is reflected in the descriptive framing of the Mechanical Properties and Quantitative Microstructural Characterisation sections (Section 3.1 and Section 3.2) and in the PCA-based multivariate backbone used in place of inferential testing. Because per-cell replication is 1–4, segmentation-derived metrics ($\varphi_{2D}$, CBSS- and pore-area distributions, oil-gland density) are reported as within-image descriptive distributions (median, IQR). Species-level comparisons are presented as descriptive rankings supplemented by PCA on the full 135-image stack, which provides a replicate-independent multivariate backbone. No species-level inferential tests are performed because the species-level sample size (n=5) is too small to support either parametric or non-parametric hypothesis testing at conventional significance thresholds.

## 3. Results and Discussion

### 3.1. Mechanical Properties

**Figure 1:**
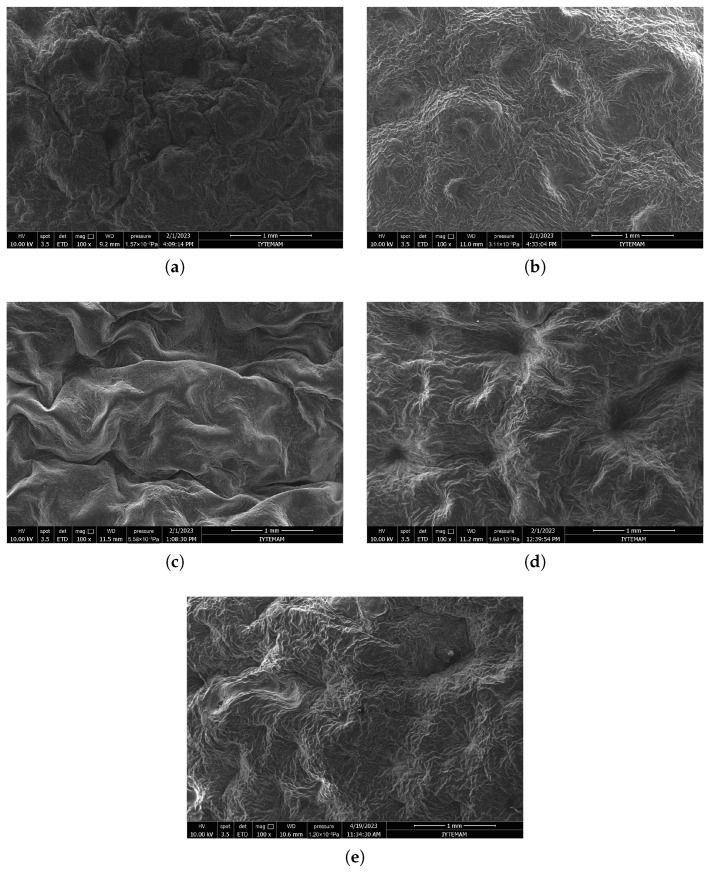
SEM micrographs of flavedo at 100×: (**a**) grapefruit, (**b**) lemon, (**c**) mandarin, (**d**) orange, and (**e**) bitter orange. Oil glands and cuticular ridges (CBSS) are visible in all flavedo images. Circular dark depressions visible across all five panels correspond to oil glands; the surrounding lighter mosaic of polygonal cells delineated by raised cuticular ridges corresponds to the cell-bounded surface segments (CBSS) analysed in Section 3.2.

**Figure 2:**
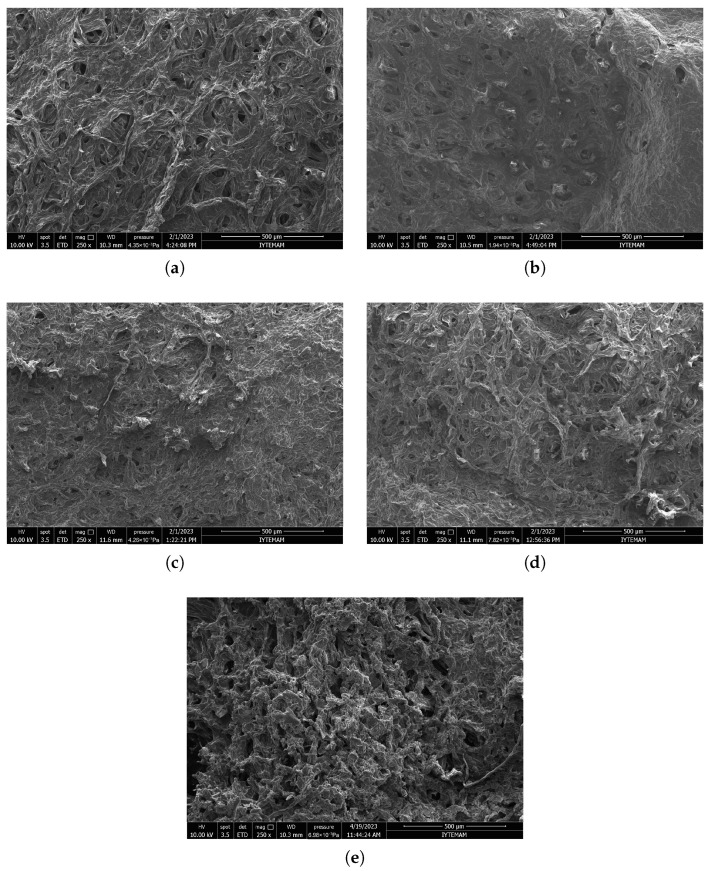
SEM micrographs of albedo at 250×: (**a**) grapefruit, (**b**) lemon, (**c**) mandarin, (**d**) orange, and (**e**) bitter orange. Large intercellular air spaces dominate the albedo of all species. Bright walls delimit the intercellular voids; the void area fraction quantified as $\varphi_{2D}$ in Table 2 corresponds to the darkest regions of each panel.

The mechanical and physical properties of the five citrus peels are summarised in Table 1. The mandarin specimen, with the thinnest peel (3.2 mm), returned the lowest values on all three tests (cutting 24.5 N, puncture 7.8 N, compression 56.9 N). The highest value on each test was distributed across three different species: the grapefruit specimen in compression (254.1 N), the bitter-orange specimen in cutting (60.8 N) and the lemon specimen in puncture (41.2 N). The differences track peel thickness and density: the mandarin peel had a density of 0.72 g/${\mathrm{cm}}^{3}$ and a thickness of 3.2 mm, whereas the grapefruit peel had 0.48 g/${\mathrm{cm}}^{3}$ and 7.2 mm. Cutting force varied broadly with peel thickness across the dataset (larger forces for thicker peels), whereas puncture and compression values did not follow thickness alone. Where the flavedo and albedo could be separated and measured with calipers, the flavedo accounted for ∼46% of the total peel thickness in the lemon specimen and ∼35% in the grapefruit and bitter-orange specimens. A larger flavedo share could in principle contribute to higher local resistance to cutting and puncture, since the flavedo is the denser layer; however, in this dataset neither the highest cutting force (bitter orange, 60.8 N; Table 1) nor the highest compression force (grapefruit, 254.1 N) belonged to the lemon specimen, so flavedo share alone does not rank the mechanical response. SEM micrographs (Figure 1 and Figure 2) show a dense, non-porous flavedo and a spongy albedo with large intercellular air spaces. The corresponding quantitative ranking of the albedo 2D void fraction $\varphi_{2D}$ measured on the exposed inner-albedo surface at 500× is lemon (36.2%) > mandarin (34.1%) > bitter orange (32.3%) > grapefruit (31.0%) > orange (28.0%), so the albedo void fraction does not align with peel or albedo density alone (full quantitative comparison is given in Section 3.2). Because each species is represented by one fruit, the values in Table 1 are best read as ordinal indicators of the five specimens, not as estimates of population means. Compression forces ranged from 56.9 N (mandarin) to 254.1 N (grapefruit), with intermediate values for bitter orange (102.2 N), orange (118.7 N) and lemon (165.8 N); the full set of cutting, puncture and compression values for all five species is given in Table 1. Probe speeds were chosen to match the dominant deformation mode of each test: 0.02 mm/s for whole-fruit compression, where slow loading approximates a quasi-static response, and 1 mm/s for cutting and puncture, where the local failure events are short-lived and a higher speed maintains a clean force signal. The compression rate used here (0.02 mm/s, equivalent to 1.2 mm/min) lies within the quasi-static loading regime previously adopted for citrus peel and related plant tissues [12,41,42]; in particular, it is comparable to the 1 mm/min quasi-static loading reported for *Citrus maxima* peel by Le Barbenchon [42].

The absolute force levels measured here are of the same order of magnitude as values previously reported for citrus peel. Quasi-static compression of five citrus species (pomelo, citron, lemon, grapefruit, orange) to 50% deformation returned median peak forces of 326, 72 and 148 N for lemon, grapefruit and orange, respectively [12]. These values are comparable in order of magnitude to our grapefruit (254.1 N) and orange (118.7 N) compression forces. Mandarin and bitter orange were not represented in that study, so the comparison is restricted to the three overlapping species. Uniaxial tensile testing of Nagpur mandarin peel reported a peel tensile strength of 0.125–0.173 MPa and a fruit firmness significantly higher along the vertical (stem–calyx) than the horizontal axis [41]; the directional sensitivity observed in that study reflects the role of local vascular-bundle orientation in the peel’s mechanical response. The combination, in mandarin, of the thinnest peel, the lowest oil-gland density and a directionally oriented flavedo film is consistent with a functionally graded interpretation of the peel [3,12]. The numerical level of the compression forces measured here differs from Jentzsch et al. [12]: our grapefruit value (254.1 N) is markedly higher than the 72 N median reported in that study, while our orange value (118.7 N) is closer to the 148 N median. Part of the difference is attributable to a different end-point: Jentzsch et al. [12] compressed to 50% deformation, whereas the present compression tests were stopped at a fixed displacement of 16 mm (25 mm for grapefruit), which for a 7.2 mm grapefruit peel corresponds to a larger fractional deformation. Specimen-to-specimen variability in ripeness, hydration and fruit size between the two studies probably also contributes. The comparison is therefore restricted to relative ordering rather than absolute force levels.

### 3.2. Quantitative Microstructural Characterisation

The SEM-derived descriptors used throughout this section have direct geometric meanings that are summarised here for the non-specialist reader. The 2D void fraction $\varphi_{2D}$ is the fraction of the imaged inner-albedo surface area occupied by intercellular air; it is measured on SEM micrographs of the exposed inner face of the peel rather than on histological cross-sections, and acts as a stereological estimator of how sponge-like the albedo is at the resolved magnification. The cell-bounded surface segment (CBSS) is the topographic projection of one cell-scale boundary on the flavedo surface, i.e., what a single epidermal cell “footprint” looks like when seen from above through the overlying wax film. It is reported here as a CBSS rather than directly as a cell because the wax film, the imaging projection and the segmentation step together prevent unambiguous identification of individual histological cells. CBSS area is the typical projected size of one such cuticular “tile” on the flavedo surface, so larger CBSS area means a coarser surface relief; the CBSS density is the number of such tiles per unit imaged surface (${\mathrm{mm}}^{-2}$) and therefore measures how tightly the relief is packed. Oil-gland density is the number of essential-oil reservoirs per ${\mathrm{mm}}^{2}$ of flavedo and oil-gland equivalent diameter is the diameter of the circle with the same projected area as one gland as seen from the flavedo surface. GLCM contrast quantifies how strongly neighbouring pixel intensities differ within a small spatial window, with higher values indicating a rougher local surface; GLCM entropy quantifies how disordered or non-repetitive the texture is, with higher values indicating a less periodic pattern [7,32,33]. The directional anisotropy index $A_{F}$ is the normalised dispersion of a GLCM feature across orientations ($0^{\circ }$, $45^{\circ }$, $90^{\circ }$, $135^{\circ }$), and so reports whether texture depends on direction or not. A large $A_{F}$ indicates an oriented pattern, analogous to the wood-grain of a sawn plank, where roughness measured along the grain differs from roughness measured across it. A small $A_{F}$ indicates an isotropic pattern, analogous to a uniformly sanded panel, where roughness is similar in all directions. Edge density is the fraction of pixels lying on a detected Canny edge in the cropped frame, and therefore measures how fine-scaled and how frequent the resolvable surface boundaries are.

#### 3.2.1. Image Inventory and Calibration Validation

All 135 micrographs were calibrated automatically; the field-of-view × magnification product was 403,273 ± 410 μm·× (relative spread ±0.10%), confirming consistent calibration across all magnifications and species. This level of internal consistency exceeds typical SEM-based food-microstructure surveys, which rely on operator-set scale-bar references [6,43].

#### 3.2.2. Flavedo Surface Topography

The flavedo surface was characterised by two complementary metric families: discrete CBSS objects at 500× (Module 4) and continuous GLCM texture at 500× and 1000× (Module 3). Structural metrics are summarised in Table 2 and texture metrics in Table 3.

CBSS area was largest on the bitter-orange flavedo (23.70 $\mu m^{2}$) and smallest on the orange flavedo (14.50 $\mu m^{2}$); circularity ordered in the opposite direction (bitter orange 0.651 vs. orange 0.748). Low circularity indicates elongated, branched cuticular ridges rather than disc-like papillae. Grapefruit showed moderate CBSS area (17.31 $\mu m^{2}$), low circularity (0.655) and the highest CBSS density (1072 ${\mathrm{mm}}^{-2}$), corresponding to a dense, branched, broadly isotropic cuticular cover. The CBSS densities reported for bitter orange and grapefruit depend on a species-specific circularity threshold (Section 2.3.8), and the absolute values should be read with this dependence in mind. To quantify this dependence, the CBSS metrics were recomputed under a uniform stricter threshold ($C_{k}\geq 0.60$) for all five species and compared with the Rule-A baseline (Table 4). Both columns of Table 4 report image-mean CBSS area and image-mean CBSS density, computed on the same 500× image stack used for Table 2, so that the Rule-A column reproduces the absolute values listed in Table 2 and the Uniform 0.60 column is directly comparable. Under the uniform 0.60 cut, mean CBSS area for bitter orange and grapefruit decreases by 21–33% and CBSS density decreases by ∼40–42%. The species ranking is preserved: bitter orange and grapefruit remain the two species with the largest CBSS area and the highest CBSS density, and lemon, mandarin and orange remain numerically indistinguishable at the smaller end. The five-species ordering of the CBSS metrics is therefore robust to the circularity threshold choice, even though the absolute values for bitter orange and grapefruit shift.

**Table 2:** Quantitative structural metrics extracted from the SEM micrographs of the five species. $\varphi_{2D}$ values are mean ± SD across 2–4 exposed inner-albedo surface images per species at 500× and a single representative inner-albedo image at 250×. CBSS area and CBSS density at 500× are reported as image-mean values across the retained 500× images per species (i.e., within-image mean area and within-image segment density per ${\mathrm{mm}}^{2}$ first computed for each micrograph, then averaged across images of the same species); the same image-mean convention is used in Table 4. Oil-gland metrics are reported at 100×. CBSS circularity is the image-mean of the per-segment Polsby–Popper circularity $C_{k}=4\pi A_{k}/P_{k}^{2}$ at 500×, averaged in the same way as CBSS area and CBSS density. Per-image distributions of CBSS area within each species are right-skewed; using the image-median in place of the image-mean of CBSS area at 500× preserves the species ranking for bitter orange (largest), grapefruit (second) and orange (smallest), while lemon and mandarin swap positions but lie within ∼1 $\mu m^{2}$ of each other under either statistic. The image-level CBSS data, including per-image medians and inter-quartile ranges, are provided in Supplementary File S1.

Species	$\varphi_{2D}$ 500× (%)	$\varphi_{2D}$ 250× (%)	Oil-Gland Dens. (${\mathrm{mm}}^{-2}$)	Oil Diam. (μm)	CBSS Area ($\mu m^{2}$)	CBSS Dens. (${\mathrm{mm}}^{-2}$)	CBSS Circ.
Lemon	36.21±1.97	32.95	1.25	46.0	15.80	550	0.746
Orange	28.03±0.47	26.83	1.70	46.2	14.50	553	0.748
Mandarin	34.13±6.39	25.32	0.63	42.2	15.15	596	0.752
Grapefruit	31.00±0.56	26.95	2.77	46.1	17.31	1072	0.655
Bitter orange	32.27±1.67	28.61	1.05	44.9	23.70	973	0.651

**Table 3:** GLCM texture metrics (contrast, entropy), GLCM directional anisotropy index $A_{F}$ and Canny edge density for the albedo and flavedo layers at 500× and 1000×.

Species	Layer	Contrast 500×	Entropy 500×	$A_{F}$ 500×	Edge Dens. 500×	Contrast 1000×	Entropy 1000×	$A_{F}$ 1000×	Edge Dens. 1000×
Lemon	albedo	13.31	10.554	0.0530	0.326	12.26	10.421	0.0408	0.283
Lemon	flavedo	18.84	11.284	0.0397	0.357	14.73	11.134	0.0312	0.353
Orange	albedo	37.35	12.100	0.0417	0.357	29.82	11.931	0.0541	0.348
Orange	flavedo	28.98	11.734	0.0459	0.374	22.58	11.446	0.0406	0.365
Mandarin	albedo	23.46	11.418	0.0707	0.356	16.45	10.973	0.0497	0.344
Mandarin	flavedo	29.37	11.698	0.0623	0.359	26.56	11.613	0.0885	0.352
Grapefruit	albedo	27.04	11.672	0.0564	0.350	19.66	11.479	0.0479	0.333
Grapefruit	flavedo	16.38	10.497	0.0358	0.364	11.75	10.354	0.0351	0.353
Bitter orange	albedo	30.31	11.760	0.0596	0.345	24.82	11.662	0.0589	0.337
Bitter orange	flavedo	14.12	11.155	0.0537	0.331	12.46	11.160	0.0466	0.317

**Table 4:** Table 4 reports a robustness check for the CBSS metrics in Table 2: the Rule-A column reproduces the baseline values used in Table 2, while the Uniform 0.60 column quantifies how those values would change under a single species-independent circularity threshold. CBSS threshold sensitivity at 500×. Values are image-mean CBSS area and image-mean CBSS density computed on the same 500× image stack as Table 2; the Rule-A column therefore matches the absolute values in Table 2. Rule-A uses the species-specific cuts described in Section 2.3.8 (bitter orange and grapefruit $C_{k}\geq 0.45$; lemon, mandarin, orange $C_{k}\geq 0.60$). The Uniform 0.60 column applies $C_{k}\geq 0.60$ to all five species. Δ values are percentage changes from Rule-A to Uniform 0.60.

Species	Mean Area Rule-A ($\mu m^{2}$)	Mean Area Unif. 0.60 ($\mu m^{2}$)	Δ Area (%)	Density Rule-A (${\mathrm{mm}}^{-2}$)	Density Unif. 0.60 (${\mathrm{mm}}^{-2}$)	Δ Density (%)
Bitter orange	23.70	15.84	−33.2	973	567	−41.8
Grapefruit	17.31	13.74	−20.7	1072	623	−41.9
Lemon	15.80	15.80	0.0	550	550	0.0
Mandarin	15.15	15.15	0.0	596	596	0.0
Orange	14.50	14.50	0.0	553	553	0.0

Mandarin occupies a different region of the same descriptor space. Its CBSS area (15.15 $\mu m^{2}$) and density (596 ${\mathrm{mm}}^{-2}$) are intermediate, but flavedo GLCM contrast at 1000× was the highest among the five fruits (26.56), and the directional anisotropy index $A_{F}$ was markedly elevated (0.0885 vs. 0.031–0.047 for the other four fruits). Values of $A_{F}\geq 0.07$ indicate clearly directional patterns, consistent with flow-aligned cuticular wax deposition. The use of GLCM contrast as a discriminative texture descriptor for citrus surfaces is supported by recent work on non-destructive classification of sweetness and firmness in oranges using CCI–GLCM-TF descriptors [34].

Two observations follow. First, citrus flavedo varies along two largely independent axes in this descriptor space: discrete relief amplitude (CBSS area, density) and directional roughness ($A_{F}$). Second, grapefruit and bitter orange are both visually “rough” but along different axes: grapefruit appears dense and broadly isotropic, whereas bitter orange is large-featured and irregular.

Qualitative inspection of the high-magnification flavedo micrographs (1000×–5000×) reveals five distinct epicuticular-wax morphologies. Lemon shows fine, thread-like cuticular wrinkles. Grapefruit shows a fragmented, plate-like cuticle with sub-micron acicular wax crystalloids on broken plate edges, consistent with its low entropy (10.354) and dense CBSS cover. Mandarin shows a continuous, plastic, undulating wax film with globular wax deposits and no resolvable fractures, consistent with the highest 1000×-flavedo contrast (26.56) and the highest 1000×-flavedo $A_{F}$ (0.0885) of the dataset, i.e., a smooth but directionally oriented film with pronounced large-scale intensity variation along the orientation of the underlying wax flow. Orange shows stacked, chunky wax platelets with sparse micro-fissures, an intermediate morphology between the grapefruit and mandarin micrographs. Bitter orange shows the most heterogeneous surface, with broad cuticular folds and locally separated wax sheets. Stomatal apertures behave consistently with these wax morphologies: mandarin stomata are sunken and occluded by the overlying wax film at 1000×–10,000×, orange stomata remain visibly open at 250×–5000×, and grapefruit and lemon stomata are obscured by either fragmented or thread-like cuticular relief. The five wax morphologies described here are consistent with the cuticular-thickness and epicuticular-wax variability reported for Fortune and Clemenules mandarins, where cuticular thickness in the range 3–5.5 μm co-varies with isolated-cuticle water permeability [44]. The fragmented, plate-like cuticle of grapefruit and the broad cuticular folds with locally separated sheets observed on bitter orange resemble the cracks and fissures around stomata that have been used as nondestructive indicators of cuticular damage in grapefruit and lemon [45]. In those species, cracks act as preferential pathways for water loss. This provides a structural rationale for treating CBSS density and continuity as candidate barrier-quality descriptors.

#### 3.2.3. Oil-Gland Geometry

Oil glands segmented at 100× in the flavedo span a 4.4-fold range in density (0.63–2.77 ${\mathrm{mm}}^{-2}$) across the five fruits, while the equivalent diameter is essentially invariant (42.2–46.2 μm). Grapefruit showed the highest gland density (2.77 ${\mathrm{mm}}^{-2}$) together with the densest CBSS cover (1072 ${\mathrm{mm}}^{-2}$): a dense cuticular cover does not preclude tight gland tiling. The five fruits do not enlarge individual glands; they tile small glands more or less tightly into the available flavedo surface. The mandarin combination of the lowest density (0.63 ${\mathrm{mm}}^{-2}$) and the smallest diameter (42.2 μm) is qualitatively consistent with the low oil yield reported for *C. reticulata*. The near-constancy of gland diameter at ∼45 μm is the most robust observation in this dataset and is directly transferable to biomimetic panel design as a target inclusion diameter.

#### 3.2.4. Albedo Internal Architecture

Albedo was characterised by the 2D void fraction $\varphi_{2D}$ computed on SEM micrographs of the exposed inner-albedo surface at 250× and 500× (Table 2) and by GLCM texture at the same magnifications (Table 3). The ranking at 500× is lemon (36.21±1.97%) > mandarin (34.13±6.39%) > bitter orange (32.27±1.67%) > grapefruit (31.00±0.56%) > orange (28.03±0.47%). At 250× the absolute values are systematically lower (range 25.3–32.9% at 250× versus 28.0–36.2% at 500×) because the lower-magnification frame includes parenchyma fragments excluded at higher magnification [29]. The species ranking shifts between the two magnifications: lemon remains the most porous at both, but mandarin drops from the second-largest $\varphi_{2D}$ at 500× (34.13%) to the smallest at 250× (25.32%), consistent with the directionally oriented mandarin flavedo film of Section 3.2.2 and with the larger 500× standard deviation reported for mandarin in Table 2. The 500× ranking is therefore preferred as the working ordinal indicator and is the one used for subsequent structure–function discussion.

$\varphi_{2D}$, measured here from the exposed inner-albedo surface, acts as a stereological estimator of the underlying $\varphi_{3D}$; under isotropy $\varphi_{2D}\approx \varphi_{3D}$, but real albedo is anisotropic and the orientation of the exposed inner surface relative to the bulk void network biases the apparent void fraction.The narrow standard deviations on the lemon, grapefruit, orange and bitter-orange micrographs (≤1.97%) indicate that the 2–4 inner-albedo surface images per fruit capture a dominant void-fraction scale within each fruit; the larger mandarin SD (6.39%) is informative—the mandarin albedo is spatially heterogeneous across the imaged inner surface, with alternating dense and loose regions, consistent with the directionally oriented flavedo film of Section 3.2.2. Similar 2D heterogeneity has been described for other porous food matrices using digital image analysis with multilevel Otsu segmentation [31].

In lemon, the high $\varphi_{2D}$ pairs with the lowest 500×-albedo GLCM entropy (10.554) and the lowest 500×-albedo contrast (13.31) of the five fruits, indicating an open and structurally simple, sponge-like albedo (large voids, few resolvable boundaries, low intensity-gradient information). In orange, the lowest $\varphi_{2D}$ pairs with the highest 500×-albedo contrast (37.35) and the highest 500×-albedo entropy (12.100), a fine-grained, high-information mesh that is structurally well-suited to bioinspired panels in which mechanical integrity outweighs permeability. A comparable inverse coupling between visible micro-cracking and bulk tensile strength has been reported in SEM-based tensile work on apple, pear, mango and aubergine peels: peels with wider and more frequent epidermal micro-cracks exhibited the lowest tensile strengths and crack-free peels the highest [46]. The structure–texture coupling described here for the citrus albedo follows the same direction.

High-magnification albedo micrographs (2500×–10,000×) reveal a secondary, species-dependent feature class: needle-like and rosette-like deposits decorating the parenchyma walls. These deposits are most prominent and well-developed on lemon albedo, as large, free-standing rosette clusters in intercellular voids. On orange albedo they are distributed as a quasi-continuous nano-needle coating. On mandarin albedo they are restricted to small encapsulated clusters trapped between collapsed fibres. They are essentially absent on grapefruit albedo, where the fibres are smooth and ribbon-like without recrystallised features. The phenomenon is consistent with crystallisation of soluble flavonoid components (predominantly hesperidin in lemon, orange and mandarin, and naringin in grapefruit) during air-drying, as reported for citrus by-product matrices [5,32]. The underlying chemistry was not quantified in the present work (no XRD/FTIR/HPLC); these deposits are reported as descriptive features only.

They were not separately segmented or masked prior to texture analysis: at the magnifications used for the quantitative metrics (500× and 1000×) the deposits were not resolvable as discrete objects, and the GLCM, anisotropy and edge-density values in Table 3 were computed on the full CLAHE-enhanced frame.

#### 3.2.5. PCA Patterns

At all four magnifications, PCA separates flavedo and albedo into two clearly distinct blocks. PC1 + PC2 explained variance: 100× = 91.8% (PC1 = 73.8%, PC2 = 18.0%); 250× = 86.2%; 500× = 86.8%; 1000× = 83.7%. The flavedo–albedo separation is preserved at every magnification, while species-specific clusters become more legible at higher magnifications. The pattern indicates that different magnifications carry complementary information: peel architecture at low magnification and surface film at high magnification. Comparable PCA-based summarisation of SEM-derived texture descriptors has been used to relate food microstructure to quality attributes in tilapia [47], supporting the use of PCA as a replicate-independent multivariate backbone for small-*n* SEM datasets.

### 3.3. Implications for Bioinspired Design

**Figure 3:**
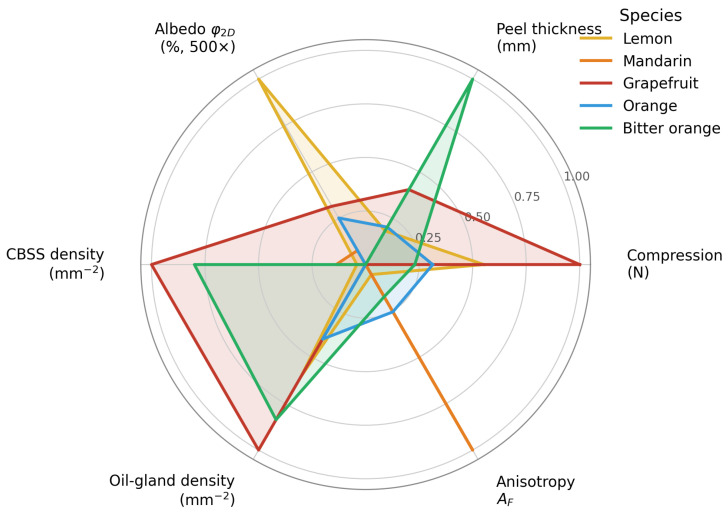
Descriptor-space radar plot of the five citrus species. Six engineering-relevant descriptors are plotted clockwise from the top: whole-fruit compression force (N), peel thickness (mm), albedo void fraction $\varphi_{2D}$ at 500× (%), CBSS density (${\mathrm{mm}}^{-2}$), oil-gland density (${\mathrm{mm}}^{-2}$) and GLCM directional anisotropy index $A_{F}$ (dimensionless). For each descriptor, the five species values are min–max normalised across the panel, so that the species attaining the largest value reaches the outer ring (1.0) and the species attaining the smallest value sits at the centre (0.0); axes are therefore non-dimensional and the plot encodes between-species rank order rather than absolute magnitudes. Mutual descriptor scales are not preserved by the normalisation, and the visual extent on each axis depends on the within-panel spread of that descriptor; absolute values, units and within-image standard deviations are given in Table 1, Table 2 and Table 3. Each closed polygon corresponds to one species (legend, top right). Because each species is represented by one specimen (Section 2), the polygons should be read as ordinal indicators of inter-species ranking and not as estimates of population means. Bitter orange peaks on peel thickness, grapefruit on compression force, CBSS density and oil-gland density, lemon on albedo porosity, and mandarin on anisotropy; orange occupies an interior position on all six axes and acts as a gap-filling reference.

The descriptor space defined by the framework separates the five fruits into a small set of candidate bioinspired-design templates whose downstream applications include but are not limited to packaging. A citrus-inspired panel does not need to copy the albedo porosity in detail. The present data suggest that it can combine two functions in a layered design: a dense and broadly isotropic CBSS-like surface relief for barrier function (grapefruit-type), and a thick layered flavedo–albedo bulk composite for mechanical absorption (bitter-orange-type, with 13.1 mm pericarp and 60.8 N cutting force in our dataset). Grapefruit illustrates that high CBSS density and high oil-gland density can coexist within the same surface, so that dual-function panels (barrier + reservoir) could in principle carry inclusions up to ∼2.8 ${\mathrm{mm}}^{-2}$ when the inclusion diameter is held near 45 μm. Grapefruit also combines the highest CBSS density (1072 ${\mathrm{mm}}^{-2}$) with the highest whole-fruit compression force in the dataset (254.1 N), so the dense CBSS cover co-occurs with a stiff macroscopic mechanical response rather than with a soft, dehydration-prone surface. This co-occurrence supports the use of grapefruit as the working surface-film template: a dense, isotropic, mechanically supported relief rather than a film inferred from surface metrics alone. The distribution of the five species across the six engineering-relevant descriptors used here is summarised in Figure 3. The bitter-orange cuticle indicates that mechanical absorption is associated with large, irregular surface features that compromise barrier uniformity; these templates therefore lend themselves naturally to a graded panel rather than to a single structure. The above transfer is, however, expressed in 2D projected terms ($\varphi_{2D}$ on the exposed inner-albedo surface, CBSS area/density on the flavedo surface). It remains subject to validation against true 3D porosity and against the volumetric oil-gland distribution. Without such validation the proposed templates should be regarded as initial design hypotheses rather than as quantitative specifications. The graded density profile implicit in the flavedo–albedo transition is quantified here as a shift in $\varphi_{2D}$, GLCM contrast and CBSS density across the two layers. It provides a spatially resolved input for multi-material or variable-infill additive manufacturing strategies, in which layer-specific mechanical targets can be assigned without requiring sub-50μm resolution at the bulk scale.

The mandarin geometry can be read as a negative template: surface anisotropy alone, without a thick layered cover and without dense CBSS, does not provide a robust barrier. The lemon albedo, with its high inner-surface void fraction and open mesh, provides a template for a lightweight, permeable, thermally insulating bulk layer that is not intended to seal moisture but to buffer mechanical and thermal load. The use of GLCM contrast as a quantitative bioinspired design parameter is supported by recent SEM-fractal reviews of food surfaces [6,7] and by analogous CCI–GLCM frameworks for orange-fruit quality [34]. The macroscopic feasibility of building objects directly from the unmodified peel has already been demonstrated for orange-peel binderless boards (W100 formulation, bulk density 558 kg/$m^{3}$, modulus of rupture 0.09 MPa, thermal conductivity 0.065–0.066 W/m·K) [17]. The descriptors reported here provide one route to refining such panels, by choosing surface (grapefruit-type) and bulk (bitter-orange or lemon-type) layers independently. The 45 μm oil-gland equivalent diameter reported here also imposes a manufacturing constraint on any direct geometric replication of the gland–CBSS mosaic. This length scale lies below the resolution of most desktop fused-deposition extrusion (typically 100–200 μm) and at the limit of consumer stereolithography (∼25–50 μm); reproduction at native dimensions therefore requires laboratory-scale processes such as two-photon polymerisation [48,49]. Practical surrogates are likely to operate at a coarser absolute length scale and to reproduce the descriptor ratios (CBSS density relative to oil-gland density; CBSS area to gland diameter) rather than the native dimensions verbatim.

### 3.4. Limitations

The following limitations should be acknowledged. (i) Plant material was sourced from a single local retailer in Izmir on the same day. Cultivar, growing region, harvest date and post-harvest history were not recorded, so all between-species differences should be read as differences between five nominally typical retail-grade specimens, not as differences between defined cultivars or production systems. (ii) SEM image-level replication is uneven (1–4 micrographs per species–layer–magnification cell), so the Section 3.2 quantitative descriptors are reported as within-image descriptive statistics; species-level inferential testing is not attempted. The species-level sample size (n=5) is also below conventional thresholds for parametric and most non-parametric tests on the structure–function relationships. All species-level comparisons in Section 3.2 are therefore framed as descriptive rankings supported by PCA on the full 135-image stack. (iii) Reported albedo porosity values are 2D void fractions $\varphi_{2D}$ computed on SEM micrographs of the exposed inner-albedo surface, not on histological cross-sections or volumetric reconstructions; they should be read as stereological estimators of the underlying 3D void fraction, and absolute volumetric porosity would require micro-CT [29]. The orientation of the manually exposed inner surface relative to the bulk albedo void network biases $\varphi_{2D}$ in anisotropic specimens (visible as the elevated mandarin SD in Table 2); a controlled sampling protocol with randomised inner-surface exposure or formal stereological correction would be required to remove this bias. (iv) SEM specimens were briefly water-rinsed, ambient air-dried only (no critical-point or freeze drying), and sputter-coated with ∼5–10 nm Au. This protocol preserves native cuticular surface features but may produce shrinkage cracks in the albedo and a thin amorphous Au layer that contributes uniformly to GLCM contrast and entropy. Because all samples were processed identically, these artefacts are expected to bias absolute metric values uniformly across species and to preserve the relative rankings on which the discussion rests. Reproduction of absolute values would require a controlled cryogenic preparation pathway. (v) Chemical identity of the rosette-like and needle-like deposits in the high-magnification albedo micrographs was not determined; the assignment to crystallised flavonoids is given as a literature-consistent hypothesis only, and identity confirmation would require XRD, FTIR or HPLC characterisation. (vi) The species-specific CBSS circularity threshold ($C_{k}\geq 0.45$ for bitter orange and grapefruit, $C_{k}\geq 0.60$ for the other three species) is an ad hoc choice based on visual inspection of segmentation overlays rather than on a pre-registered rule. To address this, a parallel CBSS analysis was performed under a single fixed threshold ($C_{k}\geq 0.60$ applied uniformly to all five species) on the same 500× image stack and reported in Table 4. The bitter-orange and grapefruit rankings (largest CBSS area, densest CBSS cover) were preserved under both threshold rules. This indicates that the species-level orderings reported in Section 3.2.2 do not depend on the threshold choice. A fully reproducible alternative (a single fixed threshold combined with shape-aware post-filtering, or a learned segmentation model) is recommended for future work.

## 4. Conclusions

A transferable quantitative framework was developed for extracting engineering-relevant descriptors from biological protective surfaces, and was demonstrated on five citrus species through coupled mechanical, gravimetric and quantitative-SEM measurements. Five species-level patterns emerge from the dataset. Grapefruit combines the highest oil-gland density (2.77 ${\mathrm{mm}}^{-2}$) with the densest CBSS cover (1072 ${\mathrm{mm}}^{-2}$) and the lowest flavedo GLCM contrast at 1000×. Bitter orange shows the largest CBSS area (23.70 $\mu m^{2}$) and the thickest pericarp (13.1 mm). Mandarin shows the most directionally oriented flavedo film ($A_{F}=0.0885$). Orange shows the smallest CBSS area (14.50 $\mu m^{2}$) together with intermediate flavedo and albedo descriptors, occupying a gap-filling position in the descriptor space. Lemon shows the most open albedo ($\varphi_{2D}=36.21\%$ at 500×). Oil-gland equivalent diameter is near-constant at ∼45 μm across the five fruits, so the species differ in how tightly they tile these glands rather than in their size, providing a geometric design constraint for bioinspired surrogates.

The principal methodological contribution is the extraction of these descriptors from 135 SEM micrographs by an automatic-calibration and segmentation pipeline with FoV × magnification consistent within ±0.10%. Principal component analysis on the same dataset separates flavedo and albedo at every magnification, and resolves species-specific clusters that become more legible at higher magnifications. The pipeline and its descriptors are openly released and are intended to be reused as a common measurement protocol for other biological protective surfaces, so that bioinspired-design studies on bark, leaves, seed coats or exoskeletons can be compared on the same quantitative basis.

From a biomimetic-design perspective, the descriptor space defined here resolves the same biological system into two distinct templates that can be transferred independently to synthetic surrogates. A surface-film template, exemplified by grapefruit, combines a dense isotropic CBSS cover with a high but compartmentalised oil-gland density at an invariant gland diameter; a cellular-core template, exemplified by bitter orange, combines a thick pericarp with a large, irregular CBSS relief and a lower-density, layered bulk. These templates supply direct numerical priors for additive-manufactured or polymer-cast biomimetic counterparts, and they isolate the parameters (gland density, CBSS area and density, $\varphi_{2D}$, GLCM contrast, $A_{F}$) that future bioinspired surfaces would have to control. The descriptors reported here are intended as ordinal between-species indicators rather than as point design targets; the numerical priors derived from them refer to the rank order and the order-of-magnitude band, and a fixed numerical specification would require multi-specimen, multi-cultivar validation beyond the scope of the present single-specimen pilot. Among the downstream applications of the surface-film template, the cuticle–CBSS–oil-gland mosaic is also the principal gas-exchange barrier of the intact fruit: the measured peel-permeability of satsuma mandarin shows a ${\mathrm{CO}}_{2}$:$O_{2}$:$N_{2}$ selectivity that favours ${\mathrm{CO}}_{2}$ retention [19], and external wax or wax–hydrocolloid coatings have been shown to lower flavedo gas permeability and to raise internal ${\mathrm{CO}}_{2}$ accumulation in mandarins and oranges [20,50]. The grapefruit-type dense, isotropic CBSS cover and the near-constant oil-gland diameter reported here therefore suggest that such native surfaces may serve as starting points for passive, MAP-compatible biomimetic films. This proposition is offered as a speculative design hypothesis and requires direct gas-permeability and shelf-life testing on engineered surrogates before any practical claim can be made. Other priorities for future work are micro-CT volumetric porosity, broader species and cultivar panels for structure–function testing, learned-segmentation validation of the SEM image-analysis pipeline, and prototyping of the surface-film and cellular-core templates in synthetic systems for direct biomimetic validation.

## Figures and Tables

**Table 1:** Physical and mechanical properties of the five citrus peels. Force values (cutting, puncture, compression) are means ± SD of five replicate measurements taken at distinct positions on a single fruit per species, and therefore describe within-specimen positional variability. Peel density is the mean ± SD of five separate fruits of the same species from the same retail batch. For each force descriptor, an indicative range of ±2 SD around the mean is given in parentheses as a descriptive guide to the spread of the five replicate positions; it is not intended as a parametric confidence interval. Peel density and thickness refer to the whole peel; albedo density was measured separately when layer separation was clean. Mandarin albedo density is reported as “–” because the thin (∼3.2 mm) mandarin peel could not be cleanly separated into flavedo and albedo for independent gravimetric measurement (see Section 2.2).

Species	Peel Dens. (g/${\mathrm{cm}}^{3}$)	Albedo Dens. (g/${\mathrm{cm}}^{3}$)	Thickness (mm)	Cutting (N)	Puncture (N)	Compression (N)
Lemon	0.68±0.03	0.39	5.0±0.42	36.3±2.74 (30.8–41.8)	41.2±3.37 (34.5–47.9)	165.8±17.17 (131–200)
Mandarin	0.72±0.06	–	3.2±0.23	24.5±3.21 (18.1–30.9)	7.8±1.14 (5.5–10.1)	56.9±7.98 (41–73)
Grapefruit	0.48±0.02	0.36	7.2±0.55	35.3±3.55 (28.2–42.4)	21.6±4.69 (12.2–31.0)	254.1±16.21 (222–286)
Orange	0.62±0.05	0.37	5.2±0.94	37.3±7.19 (22.9–51.7)	22.6±1.77 (19.1–26.1)	118.7±13.80 (91–146)
Bitter orange	0.47±0.12	0.35	13.1±3.74	60.8±16.05 (28.7–92.9)	23.5±3.00 (17.5–29.5)	102.2±13.62 (75–129)

## Data Availability

The complete SEM image stack (135 micrographs across five citrus species, three peel regions and four magnifications), the per-image quantitative outputs (M1–M4 CSV tables underlying Table 2 and Table 4) and the SEM image-analysis pipeline (v3.3) are openly available on Zenodo at https://doi.org/10.5281/zenodo.20350228. The repository contains the raw micrographs, the Python source code with a version-bounded requirements file, a worked example with synthetic input, and documentation linking each pipeline parameter to the corresponding equation in Section 2.3. Any additional data are available from the corresponding author upon reasonable request.

## Supplementary Materials

The following supporting information can be downloaded at: https://www.mdpi.com/article/10.3390/biomimetics11070451/s1, Supplementary File S1: Image-level CBSS data underlying Table 2 and Table 4, including per-image CBSS area, per-image CBSS density, per-image medians and inter-quartile ranges for each species at 500×.

## Abbreviations

The following abbreviations are used in this manuscript:

CBSS	Cell-bounded surface segment
CLAHE	Contrast Limited Adaptive Histogram Equalization
GLCM	Grey-level co-occurrence matrix
MAP	Modified atmosphere packaging
PCA	Principal component analysis
SEM	Scanning electron microscopy
