# Supplementary material for: A Transferable Quantitative Framework for Extracting Engineering-Relevant Descriptors from Biological Protective Surfaces: Intra-Specimen Descriptor Mapping of Five Citrus Peels
Source: Biomimetics (Basel). 2026 Jun 30;11(7):451. doi: 10.3390/biomimetics11070451 (PMC13406879; doi:10.3390/biomimetics11070451)
Supplement: Supplementary file 1 [file biomimetics-11-00451-s001.zip › biomimetics-4374911-supplementary.pdf]

Supplementary File S1 - Sheet 1: Per-image CBSS metrics (500x)

Image-level CBSS metrics for each 500x SEM micrograph used in Table 2 (structural) and Table 4 (CBSS sensitivity). Each row is one micrograph. Area values use the Rule-A species-specific circularity threshold (C_k) given in the last column. Citrus peel SEM dataset; magnification = 500x.

Species	Layer	Replicate	Image File	N Segments	Mean CBSS Area (um^2)	Median CBSS Area (um^2)	SD CBSS Area (um^2)	Mean Circularity	CBSS Density (mm^-2)	Circularity Threshold (C_k)
Bitter Orange	albedo	3	Turunc-Ic_500x_200Mm_03.tif	511	23,725	16,100	28,118	0,654	1141,2	0,450
Bitter Orange	albedo	10	Turunc-Ic_500x_200Mm_10.tif	573	16,357	12,938	11,950	0,656	1279,6	0,450
Bitter Orange	albedo	17	Turunc-Ic_500x_300Mm_17.tif	518	17,959	13,585	16,340	0,651	1150,6	0,450
Bitter Orange	albedo	24	Turunc-Ic_500x_300Mm_24.tif	506	20,124	15,898	12,932	0,647	1124,0	0,450
Bitter Orange	flavedo	3	Turunc-Dis_500x_200Mm_03.tif	285	35,084	19,550	43,104	0,639	636,5	0,450
Bitter Orange	flavedo	8	Turunc-Dis_500x_200Mm_08.tif	390	26,495	16,100	29,505	0,659	870,9	0,450
Bitter Orange	flavedo	11	Turunc-Dis_500x_200Mm_11.tif	272	26,124	15,956	31,674	0,655	607,4	0,450
Grapefruit	albedo	3	Greyfurt-Ic_500x_300Mm_03.tif	482	19,256	14,453	16,812	0,648	1070,6	0,450
Grapefruit	albedo	7	Greyfurt-Ic_500x_300Mm_07.tif	435	19,414	14,163	15,571	0,639	966,2	0,450
Grapefruit	flavedo	3	Greyfurt-Dis_500x_300Mm_03.tif	513	14,757	12,429	6,596	0,673	1139,5	0,450
Grapefruit	flavedo	8	Greyfurt-Dis_500x_300Mm_08.tif	500	15,823	13,296	7,726	0,659	1110,6	0,450
Lemon	albedo	5	Limon-Ic_500x_300Mm_05.tif	325	13,179	11,273	5,139	0,750	721,9	0,600
Lemon	albedo	12	Limon-Ic_500x_300Mm_12.tif	264	12,625	11,418	4,193	0,741	586,4	0,600
Lemon	flavedo	3	Limon-Dis_500x_300Mm_03.tif	221	17,029	13,296	11,001	0,750	490,9	0,600
Lemon	flavedo	7	Limon-Dis_500x_300Mm_07.tif	181	20,385	14,163	16,684	0,741	402,0	0,600
Mandarin	albedo	3	Mandalina-Ic_500x_300Mm_03.tif	250	18,117	11,851	26,036	0,756	555,3	0,600
Mandarin	albedo	4	Mandalina-Ic_500x_300Mm_04.tif	263	12,026	10,695	3,832	0,748	584,2	0,600
Mandarin	flavedo	3	Mandalina-Dis_500x_300Mm_03.tif	343	12,390	10,984	4,903	0,754	761,9	0,600
Mandarin	flavedo	9	Mandalina-Dis_500x_300Mm_09.tif	217	18,070	11,562	40,462	0,750	482,0	0,600
Orange	albedo	3	Portakal-Ic_500x_300Mm_03.tif	298	14,232	11,562	11,792	0,744	661,9	0,600
Orange	albedo	9	Portakal-Ic_500x_300Mm_09.tif	248	15,173	12,140	9,309	0,747	550,9	0,600
Orange	flavedo	3	Portakal-Dis_500x_300Mm_03.tif	255	14,893	12,140	13,482	0,741	566,4	0,600
Orange	flavedo	8	Portakal-Dis_500x_300Mm_08.tif	195	13,683	10,984	7,990	0,760	433,1	0,600

Supplementary File S1 - Sheet 2: Per-species CBSS area distribution (500x)

Distribution of individual CBSS segment areas at 500x, pooled across all retained 500x micrographs of each species. Median, first quartile (Q1), third quartile (Q3) and inter-quartile range (IQR = Q3 - Q1) summarise the right-skewed per-segment area distribution underlying the image-mean values reported in Table 2 and Table 4.

Species	N Segments (pooled)	Mean Area (um^2)	Median Area (um^2)	Q1 (25%) (um^2)	Q3 (75%) (um^2)	IQR (um^2)	SD (um^2)
Bitter Orange	3055	22,396	15,031	10,984	23,288	12,304	25,180
Grapefruit	1930	17,206	13,585	10,406	19,655	9,250	12,500
Lemon	991	15,206	11,851	9,828	16,187	6,359	9,979
Mandarin	1073	14,784	11,273	9,539	14,163	4,625	22,518
Orange	996	14,528	11,562	9,828	15,320	5,492	11,053
